# Isolation and 2,4-D-degrading characteristics of *Cupriavidus campinensis* BJ71

**DOI:** 10.1590/S1517-838246220140211

**Published:** 2015-06-01

**Authors:** Lizhen Han, Degang Zhao, Cuicui Li

**Affiliations:** 1 The Key Laboratory of Plant Resources Conservation and Germplasm Innovation in Mountainous Region, Ministry of Education, Guiyang, China, The Key Laboratory of Plant Resources Conservation and Germplasm Innovation in Mountainous Region, Ministry of Education, Guiyang, China.; 2 Guizhou University, College of Life Sciences, Guizhou University, Guiyang, China, College of Life Sciences, Guizhou University, Guiyang, China.; 3 Guizhou University, Institute of Agro-Bioengineering, Guizhou University, Guiyang, China, Institute of Agro-Bioengineering, Guizhou University, Guiyang, China.

**Keywords:** 2,4-D-degrading bacterial strain, bioremediation, *Cupriavidus campinensis* BJ71, degradation characteristics

## Abstract

An indigenous bacterial strain capable of utilizing 2,4-dichlorophenoxyacetic acid as the sole carbon and energy source was isolated from a soil used for grown wheat with a long-term history of herbicide use in Beijing, China. The strain BJ71 was identified as *Cupriavidus campinensis* based on its 16S rRNA sequence analysis and morphological, physiological, and biochemical characteristics. The degradation characteristics of strain BJ71 were evaluated. The optimal conditions for 2,4-D degradation were as follows: pH 7.0, 30 °C, 3% (v/v) inoculum size, and an initial 2,4-D concentration of 350 mg L^−1^. Up to 99.57% of the 2,4-D was degraded under optimal conditions after 6 days of incubation. Strain BJ71 was also able to degrade quizalofop and fluroxypyr. This is the first report of a 2,4-D-degrader containing *tfdA* gene that can utilize these two herbicides. In a biodegradation experiment, 87.13% and 42.53% of 2,4-D (initial concentration, 350 mg kg^−1^) was degraded in non-sterile and sterilized soil inoculated with BJ71, respectively, after 14 days. The 2,4-D degradation was more rapid in a soil microcosm including BJ71 than in a soil microcosm without BJ71. These results indicate that strain BJ71 is a potential candidate for the bioremediation of soil contaminated with the herbicide 2,4-D.

## Introduction

The post-emergence systemic herbicide 2,4-dichlorophenoxyacetic acid (2,4-D) has been widely used to control dicotyledonous weeds in cereal and grass crops for more than 60 years. Although this herbicide has a short half-life in soil and aquatic environments, its extensive use is a cause for concern because of the potential threat to the environment and human health (Chinalia *et al.*, 2007). The WHO (World Health Organization) has classified 2,4-D as a hormonal herbicide with level II toxicity. It is considered to be a carcinogenic agent affecting the liver and heart. It also affects the central nervous system, leading to convulsions (Garc'a *et al.*, 2006; Maloney and Waxman, 1999). Because of the relatively high water solubility and low soil-absorption coefficient of the free acid of 2,4-D, it often contaminates the environment when it enters streams, rivers, or lakes after drainage of agricultural lands (Chinalia and Killham, 2006; El-Bestawy and Hans-Jorgen, 2007; Lane, 1991). Most of the 2,4-D-degrading bacteria characterized to date are members of genera belonging to the β and γ subdivisions of the class Proteobacteria, and have been isolated from 2,4-D-treated environments (Gonzalez *et al.*, 2012; Kamagata *et al.*, 1997; Lee *et al.*, 2005). The best-described pathway for 2,4-D degradation is the plasmid-encoded pathway of the bacterium *Ralstonia eutropha* (formerly *Alcaligenes eutrophus*, *Waustersia eutropha*) strain JMP134 (Fukumori and Hausinger, 1993a; Fukumori and Hausinger, 1993b; Streber *et al.*, 1987). Despite the many attempts to increase the biodegradation of 2,4-D by bioaugmentation of soils with degrading strains, no significant increases in pollutant removal compared with that in uninoculated soil have been achieved (Sánchez *et al.*, 1994). The introduced strains do not always survive well in the soil environment because of various stresses, including competition with indigenous microorganisms (Manzano *et al.*, 2007; Marrón-Montiel *et al.*, 2006). For successful bioremediation of herbicide-contaminated soil, it is necessary to construct a unique niche for the desired microbes so that they can be productively exploited (Singh, 2008). Hence, the use of native 2,4-D degrading microorganisms is a feasible strategy for the bioremediation of polluted sites. The purpose of this study was to isolate an indigenous 2,4-D degrading strain using enrichment techniques, and to evaluate the degradation characteristics of the strain under different conditions. Our long-term goal is to isolate and characterize an indigenous 2,4-D degrading strain for bioaugmentation during *in situ* clean-up of sites polluted with 2,4-D in China.

## Materials and Methods

### Chemicals

2,4-D, 2-methyl-4-chloro-phenoxyacetic acid (MCPA), mecoprop (99% purity), and eosin B were purchased from Sigma-Aldrich (St. Louis, MO, USA). Ringers solution was purchased from Oxoid (Basingstoke, UK). Quizalofop, fluroxypyr, and all other chemicals were of analytical grade or higher purity.

### Enrichment and isolation of 2,4-D-degrading bacteria

Soils used for enrichment of 2,4-D-degrading bacteria were collected from wheat fields in Beijing (40°25′40″ N, 116°82′79″ E), Henan Province (34°20′34″ N, 114°15′07″ E), and Qinghai Province (36°92′15″ N, 101°67′20″ E), China. These soils have been exposed to 2,4-D for at least 10 years. Ten grams of each soil was inoculated into an Erlenmeyer flask (250 mL) containing 100 mL minimal salt medium (MSM; containing MgSO_4_ 0.2 g L^−1^, (NH_4_)_2_SO_4_ 0.5 g L^−1^, KH_2_PO_4_ 0.5 g L^−1^, K_2_HPO_4_ 1.5 g L^−1^, Na_2_EDTA 0.12 g L^−1^, NaOH 0.02 g L^−1^, ZnSO_4_ 0.004 g L^−1^, CuSO_4_ 0.001 g L^−1^, Na_2_SO_4_ 0.0001 g L^−1^, Na_2_MoO_4_ 0.001 g L^−1^, CoCl_2_ 0.0001 g L^−1^, MnSO_4_ 0.0004 g L^−1^, and 0.5 mL concentrated H_2_SO_4_, pH 7.0) (Smejkal *et al.*, 2001a), which was supplemented with 500 mg L^−1^ 2,4-D as the sole carbon and energy source. The flasks were incubated at 30 °C for 7 days in a rotary shaker at 150 rpm. Then, 10 mL enrichment culture showing degradation of 2,4-D was transferred to 100 mL fresh MSM containing 500 mg L^−1^ 2,4-D and further incubated for 7 days. Four rounds of enrichment were performed and the final enrichment cultures were serially diluted and spread on MSM plates containing 500 mg L^−1^ 2,4-D. After incubation at 30 °C for 4 days, single colonies with different morphologies were selected for further analysis of their degradation abilities. For each strain, the extent of degradation was determined by quantifying the amount of 2,4-D remaining in the culture by high-performance liquid chromatography (HPLC). One strain isolated from Beijing, designated as BJ71, degraded 2,4-D very quickly and showed the highest degradation rate of 2,4-D among the isolated strains. This strain was selected for further investigation. Isolates were stored frozen in 15% glycerol at −80 °C until analysis.

### Identification and characterization of strain BJ71

Strain BJ71 was identified based on its 16S rRNA gene sequence analysis and morphological, physiological, and biochemical tests. Morphological, physiological, and biochemical tests were conducted according to Bergey's Manual of Systematic Bacteriology (Garrity *et al.*, 2004) and related documents (Vandamme and Coenye, 2004; Vaneechoutte *et al.*, 2004). Microscopic observation was performed with an HITACHI S3400N scanning electron microscope (Hitachi, Tokyo, Japan) at 20 kV. Samples were prepared for scanning electron microscopy by fixing bacterial colonies in 2.5% glutaraldehyde solution with SEMpore (Hitachi). The samples were lyophilized and coated with gold prior to microscopic observations. Total genomic DNA was extracted with an E.Z.N.A. Bacterial DNA Kit (Omega Bio-Tek, Doraville, GA, USA) according to the manufacturer's instructions. The 16S rRNA genes were amplified from the extracted genomic DNA using the universal primers 27f (5′-AGAGTTTGATCMTGGCTCAG-3′) and 1492r (5′-TACGGYTACCTTGTTACGACTT-3′) as described previously (Lane, 1991). Each polymerase chain reaction (PCR) mixture contained 1 × Premix Taq (Ex Taq Version, Takara Bio, Shiga, Japan), 0.4 μM each primer, and ~50 ng template DNA. The PCR amplification conditions were as follows: 95 °C for 5 min; followed by 35 cycles of 95 °C for 45 s, 55 °C for 45 s, and 72 °C for 90 s. To ensure complete elongation, a final step of 72 °C for 7 min was performed. Reactions were performed in a MyCycler Thermocycler (BioRad, Hercules, CA, USA). The PCR products were sequenced by Takara (Dalian, China). The identification of phylogenetic neighbors was initially carried out using the BLASTN program against the database containing type strains with valid published prokaryotic names, acquired from the EzTaxon-e server (http://eztaxon-e.ezbiocloud.net) (Kim *et al.*, 2012). Different 16S rRNA gene sequences from GenBank were aligned using CLUSTALW version 2.0 (Larkin *et al.*, 2007). A phylogenetic tree was created using the neighbor-joining method with MEGA5.0 software (Tamura *et al.*, 2011). A bootstrap analysis based on 1000 replicates was used to place confidence estimates on the tree.

### Growth and 2,4-D-degradation conditions

Strain BJ71 was pre-cultured overnight in Luria-Bertani (LB) medium supplemented with 500 mg L^−1^ 2,4-D. The cells were harvested by centrifugation at 6,000 g for 5 min, and then washed with sterilized MSM. For all experiments, the cell concentration of the inoculum was adjusted to an OD_420_ of 0.1 (corresponding to 4.6 × 10^7^ cells mL^−1^) when inoculated into 150 mL MSM (pH 7.0) containing 2,4-D as the sole carbon source in a 250-mL Erlenmeyer flask. Flasks were incubated with shaking at 150 rpm on a rotary shaker. For controls, uninoculated media were maintained and tested in the same manner as described above. Culture samples were extracted at 24-h intervals to measure cell growth and the amount of 2,4-D remaining, as described below. We examined the effects of initial 2,4-D concentration (200, 350, 500, 650, and 800 mg L^−1^), culture temperature (22, 26, 30, and 34 °C), inoculum size (1%, 3%, 5%, 7%, and 10%), medium pH (5.0, 6.0, 7.0, 8.0, 9.0, and 10.0), and liquid medium volume (50, 100, 120, 150 mL) on cell growth and 2,4-D biodegradation. All experiments were independently performed in triplicate. Cell growth was quantified by measuring the absorbance of the sample at 420 nm using a Beckman DU640 spectrophotometer (Smejkal *et al.*, 2001b). The degradation rate was analyzed by determining the amount of 2,4-D remaining in the culture medium by HPLC. We used a Waters 600 HPLC system equipped with a C18 reverse-phase column with methanol − 0.1% H_3_PO_4_ (60:40) as the eluent. A decrease in the value of A_230_ at an elution time of ~5.5 min indicated 2,4-D metabolism. A standard curve of 2,4-D was used to calculate the percentage of degradation. Cultures were preprocessed according to the method of Suwa *et al.* (1996).

### Detection of substrate degradation by strain BJ71

Strain BJ71 was precultured to an optical density of 0.6–1.0 (OD_420_) and then serially diluted in 1/4 Ringers solution. The 10^−5^ dilution of this culture was plated onto modified Loos agar plates containing (NH_4_)_2_SO_4_ 0.1 g L^−1^, eosin B 0.04 g L^−1^, yeast extract 0.25 g L^−1^, Na_2_EDTA 0.12 g L^−1^, NaOH 0.02 g L^−1^, ZnSO_4_ 0.004 g L^−1^, CuSO_4_ 0.001 g L^−1^, Na_2_SO_4_ 0.0001 g L^−1^, Na_2_MoO_4_ 0.001 g L^−1^, CoCl_2_ 0.0001 g L^−1^, MnSO_4_ 0.0004 g L^−1^, 0.5 mL H_2_SO_4_, and 10 mL 1% (w/v) alkaline methylene blue solution. Separate carbon sources (MCPA, mecoprop, quizalofop, or fluroxypyr) were added at concentrations of 3 mmol L^−1^. Plates were set up in triplicate and incubated for 4 days at 25 °C. This method was described by Smejkal *et al.* (2001b; 2003).

### Soil microcosm degradation experiments

Organic agricultural topsoil, classified as a loamy soil with 14% clay, 32% silt, 54% sand, and 2.34% carbon (pH 6.9) was collected from a farm (N 26°25′59″ E 106°40′16″) (Guizhou, China), and then sieved (pore size, 2 mm) and air dried. The soil had not been exposed to 2,4-D previously. Soil (200 g) was added to 500-mL wide-mouth glass jars covered with aluminum foil. Control soils were sterilized by autoclaving (121 °C, 20 min) on 3 different days before starting the microcosm experiments. The soil used in the microcosm experiments was supplemented with 2,4-D at a final concentration of 350 mg kg^−1^. Strain BJ71 was grown at 30 °C in LB with 2,4-D (500 mg L^−1^), harvested by centrifugation, washed twice, and then resuspended in sodium phosphate buffer. The soil samples were incubated for 1 day to equilibrate, and then inoculated with freshly grown cultures at a cell density of 7 × 10^7^ cfu g^−1^ soil. Sterilized controls containing the same amount of 2,4-D were run simultaneously under identical conditions. After all amendments were added, the moisture content of soil was adjusted with water to 75% of the water-holding capacity. The soil was shaken every week to enhance O_2_ availability. Soil microcosms were incubated at 30 °C in the dark to prevent photodegradation of 2,4-D during the 42-day experiment. All experiments were conducted with three replicates. The concentration of 2,4-D in the soils was determined by HPLC according to the method of Holben *et al.* (1992).

### Statistical analysis

Data were analyzed using one-way analysis of variance and multiple comparisons were performed with Duncan's multiple range test using SPSS software for Windows (version 19.0).

## Results

### Isolation and screening of 2,4-D-degrading bacterial strains

We isolated 22 strains of 2,4-D-degrading bacteria by picking colonies with differing morphologies. All the strains were able to utilize 2,4-D as the sole carbon and energy source. Of the 22 strains, 13 degraded more than 60% of the initial 2,4-D (500 mg L^−1^) during 1 week of incubation at 30 °C. Sequence analyses showed that the genomes of these strains contained the conserved sequence of a class I *tfdA* gene (Han *et al.*, 2014). The BJ71 strain accumulated the largest biomass and showed the most complete degradation of 2,4-D among these 2,4-D-degrading strains. Therefore, BJ71 was selected for further investigation.

### Identification of strain BJ71

**Figure 1:**
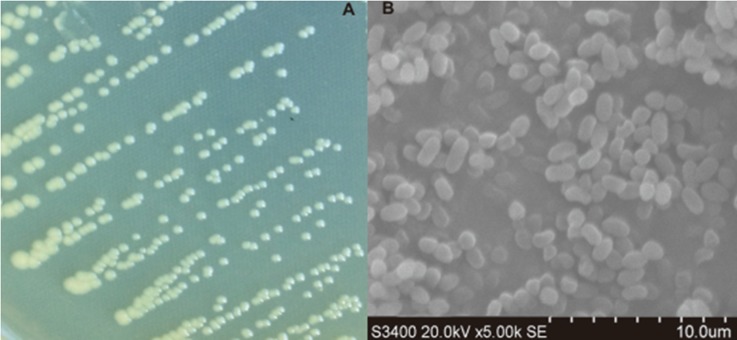
Colonies and cells of strain BJ71. (A) Photograph of BJ71 colonies on LB agar plate (B) Scanning electron micrograph of BJ71 cells.

**Figure 2:**
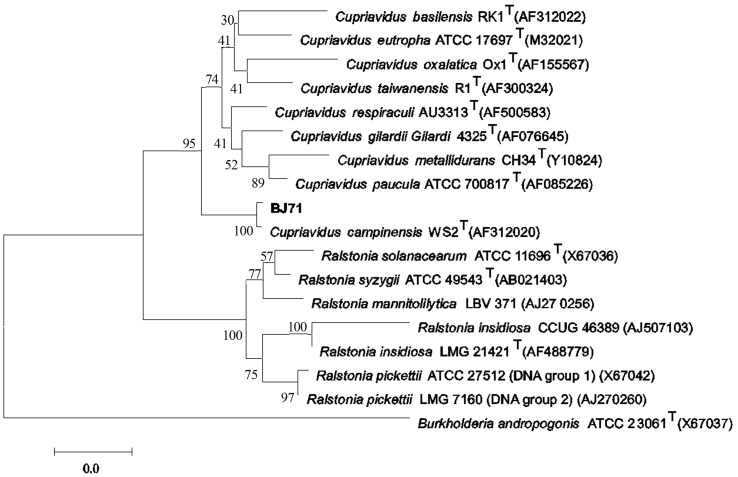
NJ tree based on 16S rRNA gene (1,390 bp) sequences showing the phylogenetic relationship between strain BJ71 and other related species in genera *Ralstonia* and *Wautersia* sp. GenBank accession number are in parentheses. Bootstrap values are based on 1,000 resampled datasets. All major branches were conserved in the maximum-parsimony tree. *Burkholderia andropogoni* ATCC 23061^T^ (X67037) served as the outgroup. Scale bar indicates number of nucleotide changes per base position analyzed.

According to the observation of cell morphology and physiological and biochemicala tests, BJ71 is a motile, Gram-negative rod-shaped bacterium that forms opalescent colonies (Figure 1) with oxidase and catalase activities. The bacterium showed positive results in urease and nitrate reduction tests, but negative results in tests for glucose fermentation and indole and citrate utilization. The 16S rRNA sequence of strain BJ71 (1,390 bp) was compared with bacterial 16S rRNA sequences in GenBank. Phylogenetic neighbors were identified by BLASTN searches against the database containing type strains with valid published prokaryotic names, acquired from the EzTaxon-e server. The 16S rRNA gene sequence of strain BJ71 showed 100% similarity to that of *Cupriavidus campinensis* WS2^T^ (GenBank accession nos. AF312020) (Figure 2). *C. campinensis* is a new species assigned in 2001. A metal-resistant type strain, WS2^T^, was isolated from a zinc-desertified area in Belgium (Goris *et al.*, 2001).

Based on its 16S rRNA gene sequence analysis and its morphological, physiological, and biochemical characteristics, strain BJ71 was identified as *Cupriavidus campinensis.* The nucleotide sequence of the 16S rRNA gene of strain BJ71 has been deposited in GenBank under the accession number KF997830.

### Effect of environmental factors on growth and 2,4-D degradation of strain BJ71

**Figure 3:**
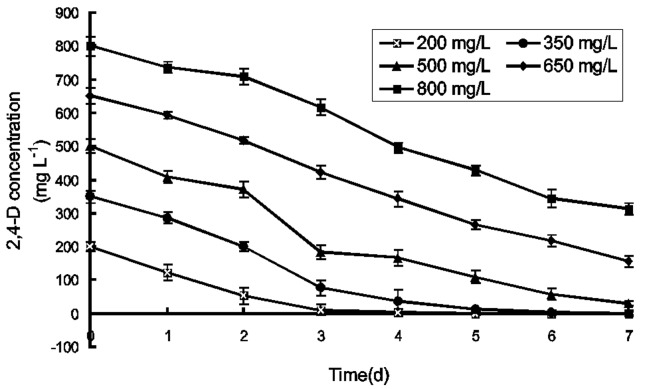
Effect of initial 2,4-D concentration on degradation of 2,4-D by strain BJ71. Incubation conditions were as follows: pH 7.0, final cell density at 420 nm adjusted to 0.1. Cells were inoculated into 150 mL MSM (pH 7.0) in a 250-mL flask at 30 °C and grown with shaking at 150 rpm on a rotary shaker. Standard errors are within 5% of the mean.

**Figure 4:**
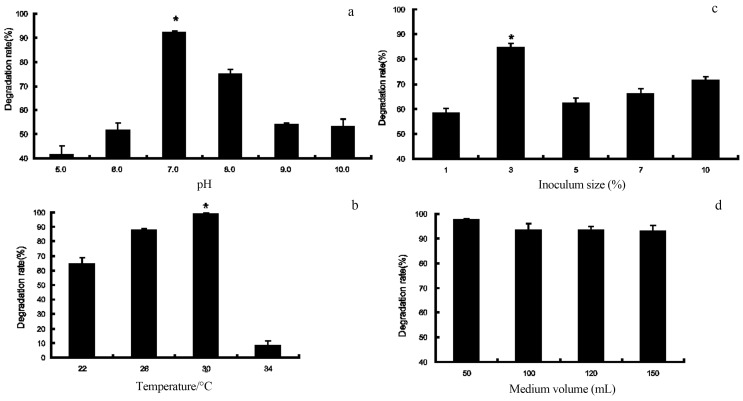
Effects of environmental conditions on degradation rate of 2,4-D by strain BJ71. Effects of pH (a), temperature (b), inoculum size (c), and medium volume (d) on 2,4-D degradation. For all experiments, final density of cells at 420 nm was adjusted to 0.1. Cells were inoculated into 150 mL MSM (pH 7.0) containing 350 mg L^−1^ 2,4-D in a 250-mL Erlenmeyer flask before incubation at 30 °C with shaking at 150 rpm, unless otherwise stated. All data were obtained at 120 h of incubation. Values shown are means of duplicate experiments. Error bars indicate standard deviation (SD). * indicates significant difference (Duncan's test, p < 0.05).

We investigated the effect of different initial 2,4-D concentrations on 2,4-D degradation by strain BJ71 (Figure 3). Strain BJ71 degraded 2,4-D quickly at low substrate concentrations (200 and 350 mg L^−1^), and showed almost complete degradation of 200 mg L^−1^ 2,4-D in 3 days. When the initial 2,4-D concentration was 350 mg L^−1^, BJ71 degraded 90% of the 2,4-D after 4 days. BJ71 was also able to degrade 2,4-D at high concentrations in MSM (94.08% of 500 mg L^−1^ and 61% of 800 mg L^−1^ after 7 days of incubation). We also investigated the effects of other factors on the 2,4-D degradation rate of strain BJ71 (Figure 4). Among six different pHs, pH 7.0 was optimal for 2,4-D degradation by BJ71. This strain was well-adapted to a wide range of pHs and was able to degrade up to 40% of the 2,4-D at pH 5.0–10.0. Temperature is a major environmental factor affecting degradation. The biodegradation rate was approximately 8.49% at the highest temperature tested (34 °C), and 98.98% at 30 °C. The optimum inoculum size was 3.0%. The degradation rate decreased when the inoculum size was too low or too high because of insufficient inoculum or nutrient limitation, respectively. There was no significant effect of different medium volumes on the 2,4-D degradation rate.

**Figure 5:**
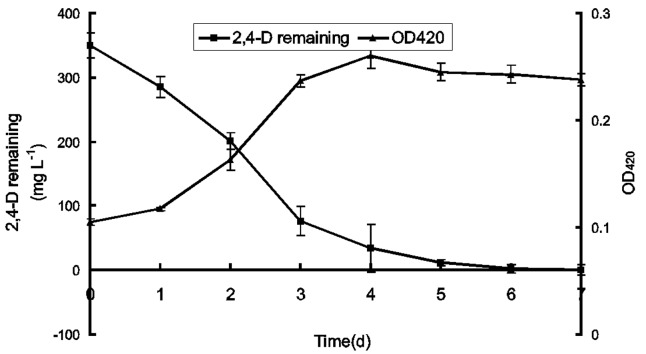
Amount of 2,4-D remaining and cell growth during degradation of 2,4-D by strain BJ71. Incubation conditions were as follows: pH 7.0, final cell density at 420 nm adjusted to 0.1. Cells were inoculated into 150 mL MSM (pH 7.0) containing 350 mg L^−1^ 2,4-D in a 250-mL Erlenmeyer flask at 30 °C and shaker at 150 rpm. Standard errors are within 5% of the mean.

The optimal conditions for 2,4-D biodegradation by strain BJ71 were as follows: initial pH of 7.0, incubation temperature of 30 °C, inoculum size of 3.0% (v/v), and an initial 2,4-D concentration of 350 mg L^−1^. The typical growth of strain BJ71 was consistent with the degradation curves under optimal conditions (Figure 5). Up to 99.57% of the 2,4-D was degraded by strain BJ71 under these optimum conditions after 6 days of incubation.

### Analysis of substrate range

It has been reported that growth on Modified Loos medium can reveal microbial dehalogenation of herbicide compounds, during which there is a cleavage of the aromatic ring (Smejkal *et al.*, 2003). BJ71 was grown on this medium supplemented with 3 mmol of several other herbicides including mecoprop, MCPA, quizalofop, and fluroxypyr. After 4 days of incubation, dark violet colonies were observed on the plates of Modified Loos medium containing 2,4-D, quizalofop and fluroxypyr. Therefore, BJ71 was able to grow on media containing the herbicides quizalofop and fluroxypyr, but not on media containing MCPA or mecoprop.

### Degradation of 2,4-D in soil

**Figure 6:**
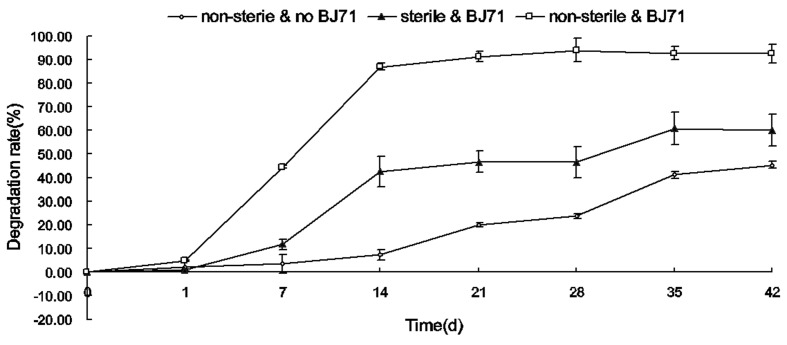
Degradation of 350 mg kg^−1^ 2,4-D in soil inoculated with strain BJ71. Soil in microcosm degradation experiments contained 2,4-D at 350 mg L^−1^, and was inoculated with BJ71 at a cell density of 7 × 10^7^ cfu g^−1^ soil. Soil microcosms were incubated at 30 °C for 42 days in darkness to prevent photodegradation of 2,4-D. Values shown are the mean of three replicates. Standard errors are within 5% of mean value.

We evaluated the patterns of degradation in non-sterilized and sterilized soils, with and without strain BJ71 (Figure 6). In non-sterile soil inoculated with strain BJ71, the degradation rate was markedly higher than in the other treatments after 42 days of incubation at 30 °C. After 14 days of incubation, 87.13% and 42.53% of 2,4-D (initial concentration of 350 mg kg^−1^) were degraded in non-sterile and sterilized soil inoculated with the strain BJ71, respectively. In non-sterile control without strain BJ71, the removal rate was only 7.34%. There was no degradation of 2,4-D in control sterilized soil, which contained 350 mg kg^−1^ 2,4-D but no inoculant (data not shown). The decrease in 2,4-D concentrations in soil lacking BJ71 indicated that other indigenous microorganisms showed some 2,4-D degrading ability. Strain BJ71 enhanced the degradation of 2,4-D when present with other indigenous microorganisms in the soil. The isolated *C. campinensis* BJ71 strain has been deposited in the China Centre for Type Culture Collection (Accession number CCTCC M 2014006).

## Discussion

In this study, we isolated an indigenous 2,4-D degrading bacterium from a polluted wheat field in Beijing, China. The strain was identified as *C. campinensis* based on its 16S rRNA gene sequence analysis and its physiological features. In previous studies, several types of 2,4-D-degrading bacteria were isolated from 2,4-D-treated environments. These bacteria have been categorized into three groups based on their degradation enzymes and physiological properties. The first group consists of copiotrophic bacteria belonging to the β and γ subdivisions of the class Proteobacteria, and includes species in the genera *Achromobacter*, *Burkholderia*, *Delftia*, *Halomonas*, *Pseudomonas*, *Ralstonia*, *Rhodoferax*, and *Variovorax*. Their 2,4-D-catabolizing enzymes are encoded by genes homologous to *tfdA*, which encodes an Fe(II)/α-ketoglutarate-dependent dioxygenase that converts 2,4-D into 2,4-dichlorophenol. The *tfdA* gene is initially found in *R. eutropha* JMP134 (Gonzalez *et al.*, 201; Lee *et al.*, 2005, Vallaeys *et al.*, 1999). The second group consists of members of the α-Proteobacteria, closely related to *Bradyrhizobium* sp. (Itoh *et al.*, 2000; Kamagata *et al.*, 1997). Members of the third group are copiotrophic α-Proteobacteria in the genus *Sphingomonas* (McGowan *et al.*, 1998; Suwa *et al.*, 1996). These strains, which include *Delftia acidovorans* MC1, *Rhodoferax* sp. P230, and *Sphingobium herbicidovorans* MH, contain *sdpA* or *rdpA* genes, which encode products that can cleave enantiomers of racemic compounds (Mller *et al.*, 2006; Schleinitz *et al.*, 2004; Westendorf *et al.*, 2006). These 2,4-D-degrading isolates have been well characterized in previous studies. The degradation capacity of strain BJ71 was greater than those reported for *Sphingomonas agrestis* strain 58-1 (Shimojo *et al.*, 2009) and three other strains; *Burkholderia cepacia* DS-1, *Pseudomonas* sp. DS-2, and *Sphingomonas paucimobilis* DS-3 (Cycon *et al.*, 2011). The 2,4-D degradation ability of strain BJ71 in medium containing 2,4-D was comparable to those reported for *C. pampae* CPDB6^T^ (Cuadrado *et al.*, 2010) and *Halomonadaceae* sp. I-18 (Maltseva *et al.*, 1996). The 2,4-D degradation efficiency of strain BJ71 was greater than that of *C. necator* JMP134, the most intensively studied chloroaromatic-degrading microorganism to date. When grown on medium containing 250 mg L^−1^ of 2,4-D as the sole carbon and energy source, JMP134 showed a maximum 2,4-D degradation rate of 70% after 10 days of incubation (Lerch *et al.*, 2007). A *Delftia* species isolated from a polluted river in Argentina was able to degrade 200 mg L^−1^ of 2,4-D in 28 h and remove 99.0% of the pollutant, whereas *C. necator* EMA-G isolated from agricultural soil in Argentina degraded 250 mg L^−1^ 2,4-D in less than 1 day (Gonzale *et al.*, 2012; Zabaloy and Gómez, 2013). *B. cepacia* YK-2 and a strain of *P. putida* isolated from the Jordan Valley completely degraded 500 mg L^−1^ 2,4-D within 28 and 45 h, respectively (Cho *et al.*, 2002; Khalil, 2003). The 2,4-D degradation efficiency of these strains in shaking culture with higher concentrations of 2,4-D or in soil microcosms was not reported. In this study, BJ71 in liquid culture showed a degradation rate of up to 60% after 7 days of incubation when grown on medium containing 800 mg L^−1^ 2,4-D.

Many 2,4-D-degrading strains do not have wide substrate specificity for chlorophenoxyalkanoic herbicides, and are limited to 2,4-D as a growth substrate (Hoffman *et al.*, 1996; Ka *et al.*, 1994). We screened an expanded set of structurally related herbicides, and unexpectedly found that this 2,4-D-degrading *C. campinensis* BJ71 strain could also degrade quizalofop and fluroxypyr. The mechanism of action of these herbicides is entirely different from that of 2,4-D. Quizalofop is in the aryloxyphenoxypropionate (AOPP) class of potent grass-selective herbicides, and fluroxypyr consists of pyridyloxyacetate compounds. Wright *et al.* (2010) reported that AAD-1 (synonymous with RdpA in *S. herbicidivorans*) has the unique ability to enantioselectively cleave R-quizalofop, and that AAD-12, (synonymous with SdpA from *D. acidovorans*) can degrade fluroxypyr. However, there have been no other reports in the literature of 2,4-D-degraders containing the *tfdA* gene that are able to degrade fluroxypyr and quizalofop. Recently, we cloned the entire *tfdA* gene of *C. campinensis* strain BJ71 (GenBank accession number: KJ028765). The sequence of the *tfdA* gene from *C. campinensis* BJ71 shows some differences from that of *C. necator* JMP134 (Han *et al.*, 2015). To our knowledge, this is the first report of a 2,4-D degrader containing the *tfdA* that can degrade several different synthetic auxins (2,4-D, fluroxypyr, and the aryloxyphenoxypropionate herbicide quizalofop). Because of these characteristics, this strain has great potential for bioremediation of these herbicides in polluted environments.

For successful bioremediation, it is important to identify and isolate appropriate microbial strains, and to ensure their survival and activity after their release into the target habitat. A key factor in the failure of bioaugmentation strategies is the rapid decline of the population of introduced cells. The introduced strain may face intense competition, predation, or parasitism in the natural environment. The most suitable candidate for bioaugmentation is an autochthonous strain that is able to survive long-term in the target habitat, and continuously degrade the contaminant (Ramos *et al.*, 1991; Thompson *et al.*, 2005). It has been reported that the batch culture method used to enrich and isolate bacteria is highly selective for fast-growing copiotrophic microbes, and that the isolation of *Cupriavidus* sp. from the most dilute positive MPN tubes may reflect its dominance in the 2,4-D degrading community in soil (Macur *et al.*, 2007; Zabaloy and Gómez, 2013). Our results show that *Cupriavidus* sp. BJ71, isolated from a polluted wheat field in China, can rapidly degrade 2,4-D in liquid medium. The biodegradation of 2,4-D was much more rapid in 2,4-D (350 mg kg^−1^)-treated soil inoculated with BJ71 than in soil without this strain. These results suggest that *C. campinensis* BJ71 could be a suitable candidate for bioaugmentation for *in situ* clean-up strategies. Because it is an indigenous organism, it is more likely to survive than are other non-indigenous bacteria when introduced into polluted soil and water environments in China.
